# The Basking Shark

**Published:** 1874-11

**Authors:** 


### THE BASKING SHARK.

An interesting ichthyological discovery has lately been made by Prof. Steenstrup, of Copenhagen. He finds that certain comb-like bodies, which have been supposed to be appendages of the skin of certain sharks, are really shifting organs appended to the interior of the gill apertures of the basking shark; and he infers that this fish, the largest shark of the northern regions, which attains a length of thirty-five feet or more, lives, like the still more gigantic whales, upon the bodies of small marine animals strained from the water by these peculiar fringes. The very fine rays composing the fringes are five or six inches long, and, were some years ago, shown by Prof. Hanover to consist of dentine, so that each of them may be regarded as, to a certain extent, the analogue of a tooth. It is remarkable that Bishop Gunnerus, who originally described the basking shark (*selachus maximus*), and regarded it as the fish that swallowed the Prophet Jonah, noticed the existence of these branchial sieves more than a century ago.

